# Knockdown of long non-coding RNA Taurine Up-Regulated 1 inhibited doxorubicin resistance of bladder urothelial carcinoma via Wnt/β-catenin pathway

**DOI:** 10.18632/oncotarget.20927

**Published:** 2017-09-15

**Authors:** Dalong Xie, Hui Zhang, Xuanhao Hu, Chao Shang

**Affiliations:** ^1^ Department of Anatomy, College of Basic Medicine, China Medical University, Shenyang, 110001, China; ^2^ Department of Urinary surgery, Shengjing Hospital, China Medical University, Shenyang, 110004, China; ^3^ Department of Neurobiology, College of Basic Medicine, China Medical University, Shenyang, 110001, China

**Keywords:** bladder urothelial carcinoma, long noncoding RNA, Taurine Up-Regulated 1, chemotherapy, Wnt/β-catenin pathway

## Abstract

In genitourinary system, bladder cancer (BC) is the most common and lethal malignant tumor, which most common type is bladder urothelial carcinoma (BUC). Long non-coding RNA (lncRNA) Taurine Up-Regulated 1 (TUG1) gene is high-expressed in several malignant tumors, including BC. In this study, over-expression of TUG1 was found in BUC tissues and cell line resistant to doxorubicin (Dox). Knockdown of TUG1 inhibited the Dox resistance and promoted the cytotoxicity induced by Dox in T24/Dox cells. TUG1 knockdown also depressed the Wnt/β-catenin pathway, and the activation the Wnt/β-catenin pathway partly reversed the inhibitory effects of TUG1 knockdown on Dox resistance in T24/Dox cells. In conclusion, up-regulation of lncRNA TUG1 was related with the poor response of BUC patients to Dox chemotherapy, knockdown of TUG1 inhibited the Dox resistance of BUC cells via Wnt/β-catenin pathway. These findings might assist in the discovery of novel potential diagnostic and therapeutic target for BUC, thereby improve the effects of clinical treatment in patients.

## INTRODUCTION

In genitourinary system, bladder cancer (BC) is the most common and lethal malignant tumor, which most common type is bladder urothelial carcinoma (BUC). Surgical operation is the preferred and key preference for BUC patients, systemic and intravesical chemotherapy may decrease BUC cell metastasis and improve patient survival [1]. Although tremendously chemotherapy strategies have been improved recently, but resistance to chemotherapeutics has severely limited the efficacies of these drugs in clinical BUC applications, the prognosis of BUC patients is still poor [2]. It is crucial to elucidate the mechanisms underlying BUC chemoresistance and identifying novel therapeutic targets will be crucial to further improvements in BUC patient prognosis.

Accumulating evidences showed that the long non-coding RNAs (lncRNAs) might play important roles in carcinogenesis [3, 4]. LncRNAs are a class of non-protein coding transcripts longer than 200 nucleotides and participated in the process of epigenetic regulation, transcriptional regulation, and posttranscriptional regulation [5–7]. With the development of bioinformatics and functional genomics studies, many lncRNAs were discovered reflecting disease progression and serving as a predictor of patient outcomes [8, 9].

Taurine Up-Regulated 1 (TUG1) gene, a novel lncRNA mapping to 22q12.2, was originally identified in retinal development by *Young TL* et al. at 2005 [10]. Recent literatures reported that TUG1 was high-expressed in several malignant tumors, including gastric cancer, colorectal cancer and pancreatic cancer, which indicated TUG1 acted as an oncogene in these tumors [11–15]. TUG1 was also up-regulated in BUC, and may be a prognostic biomarker for BUC [16, 17]. These founding suggested that TUG1 might be involved in genesis of BUC. Nevertheless, the functional roles and regulatory mechanisms of TUG1 in chemoresistance remain unknown, especially to Dox.

In this study, we emphasized the pivotal roles and regulatory mechanism of TUG1 on chemoresistance in BUC, which might helpful to improve the effects of clinical treatment in BUC.

## RESULTS

### Up-regulation of TUG1 was correlated with doxorubicin resistance of BUC

**Figure 1:**
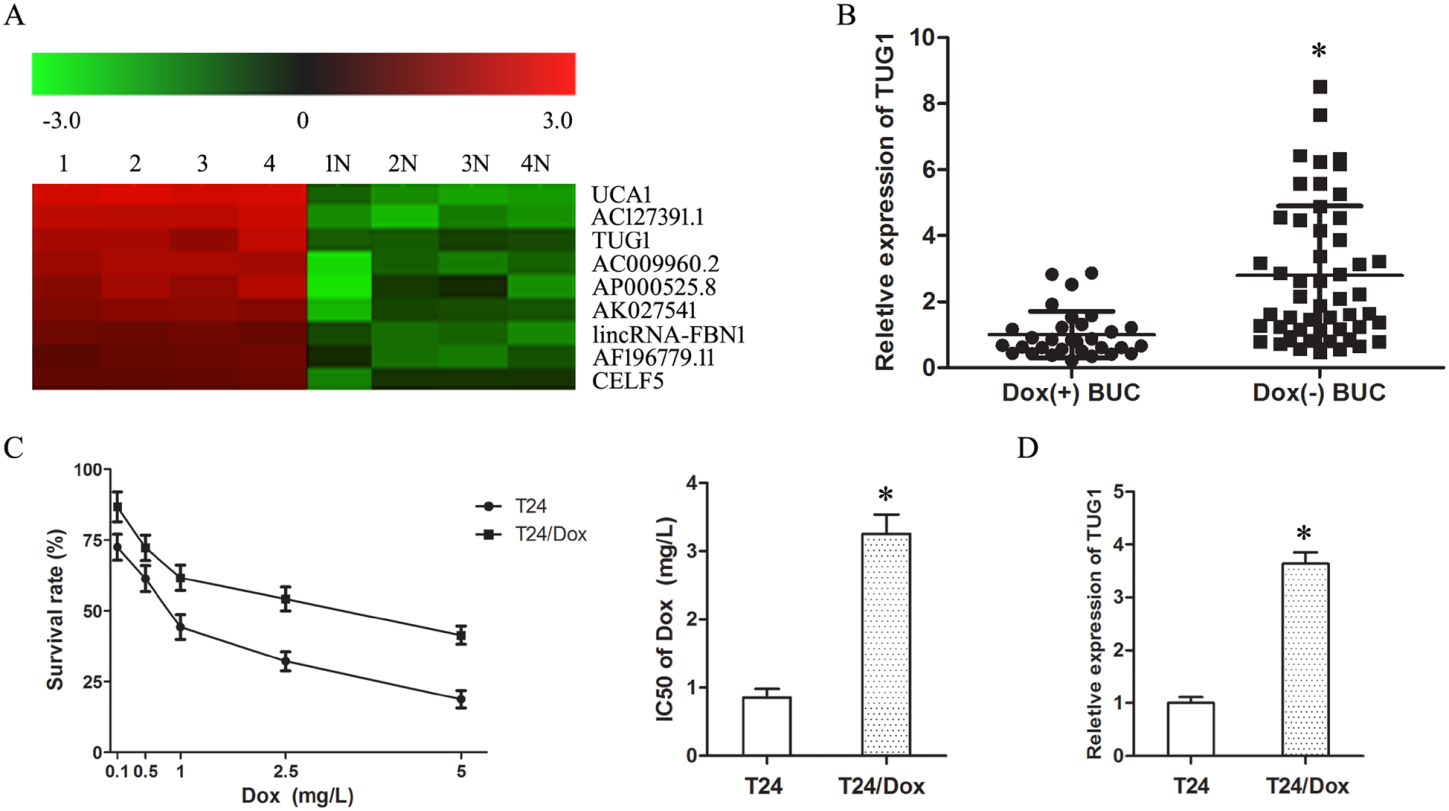
Up-regulation of TUG1 was correlated with doxorubicin resistance of BUC (**A**) LncRNA microarray analysis of total RNA isolated from Dox (−) and Dox(+) BUC samples. 1–4: Dox (−) BUC samples, 1N-4N: Dox(+) BUC samples. (**B**) The expression of TUG1 in BUC patients treated with Dox. (**C**) The dose-response curve and IC50 of T24 cells and T24/Dox cells to Dox. (**D**) The TUG1 expression in T24 cells and T24/Dox cells. *P < 0.05.

To screen the lncRNAs associated with Dox resistance in BUC, the 4 Dox (−) and 4 Dox (+) BUC samples were examined with LncRNA array. In the array, TUG1 showed an over seven-fold up-regulation in Dox (−) BUC samples compared with Dox (+) BUC samples (Figure 1A). In addition, the up-regulation of TUG1 in Dox (−) BUC samples compared with Dox (+) BUC samples was confirmed by the following large sample qRT-PCR assay (Figure 1B).

The half maximal inhibitory concentration (IC50) of T24 cells and T24/Dox cells to Dox was 0.85 ± 0.13 mg/L to 3.26 ± 0.28 mg/L. T24/Dox cells showed more powerful resistance to DOX than T24 cells, which resistance index (RI) was 3.84 (Figure 1C, *P* < 0.05). And, the expression of TUG1 in T24/Dox cells was much higher than that in T24 cells (Figure 1D, *P* < 0.05).

These results revealed that the up-regulation of TUG1 was related with the poor response of BUC patients to Dox chemotherapy, which provided initial evidence that TUG1 played a key role in the Dox resistance of BUC.

### Knockdown of TUG1 inhibited Dox resistance in T24/Dox cells

**Figure 2:**
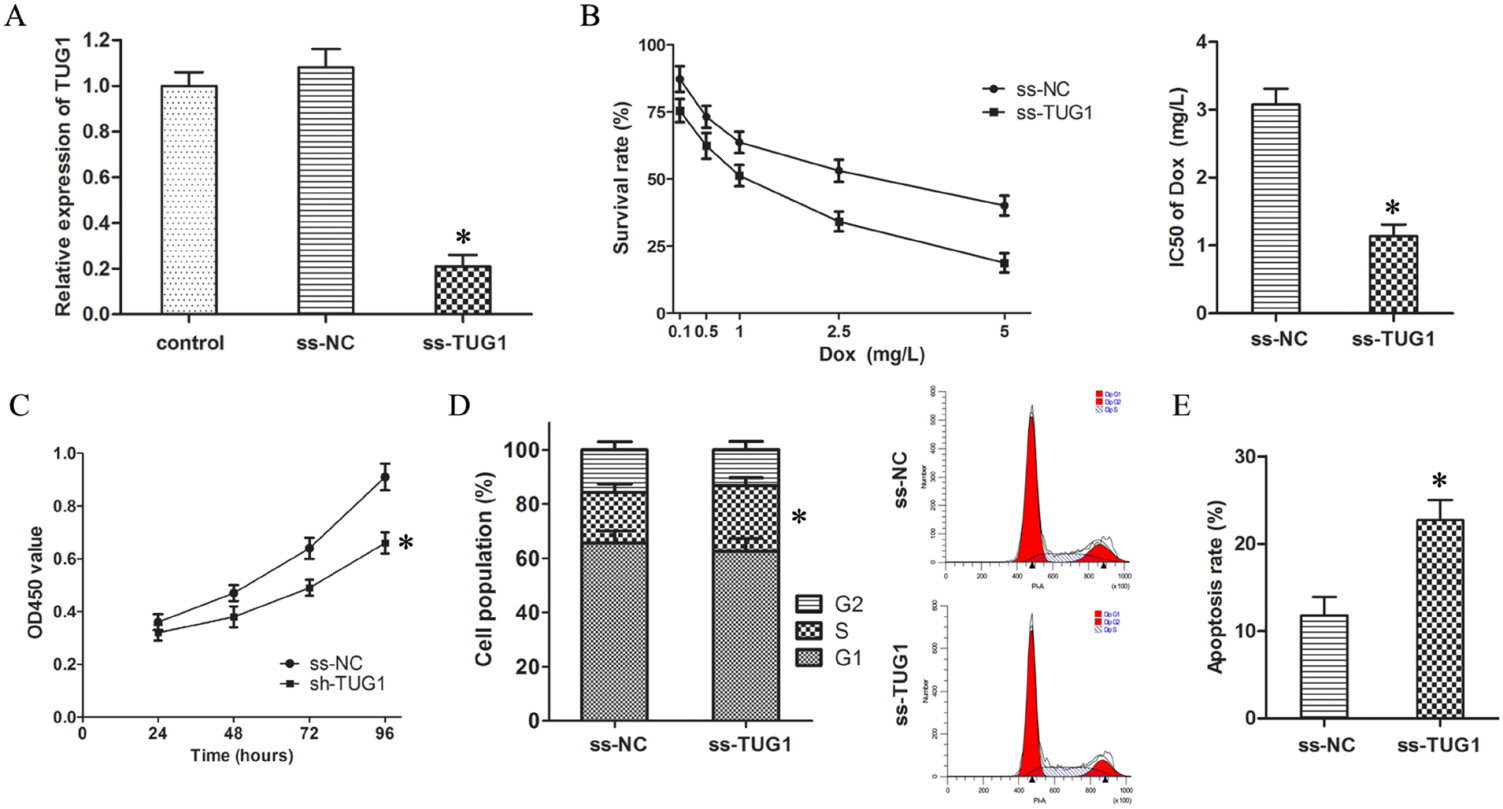
Knockdown of TUG1 inhibited Dox resistance in T24/Dox cells (**A**) The TUG1 expression in T24/Dox cells after transfection with ss-TUG1. (**B**) The dose-response curve and IC50 of T24/Dox cells to DOX. (**C**) The cell viability of T24/Dox cells detected by CCK8 assay. (**D**) The cell cycle of T24/Dox cells detected by flow cytometry. (**E**) The cell apoptosis of T24/Dox cells detected by flow cytometry. *P < 0.05.

In order to validate the vital roles of TUG1 on Dox resistance, ss-TUG1 was transfected into T24/Dox cells to knockdown the expression of TUG1 (Figure 2A).

The CCK8 assay showed knockdown of TUG1 inhibited the IC50 of T24/Dox cells to Dox from 3.08 ± 0.23 mg/L to 1.14 ± 0.17 mg/L (Figure 2B, *P* < 0.05), which demonstrated that knockdown of TUG1 depressed Dox resistance in T24/Dox cells.

Furthermore, the T24/Dox cells were treated with Dox (0.5 mg/L), and the impacts of TUG1 knockdown on the Dox-induced cytotoxicity were detected. Firstly, knockdown of TUG1 restrained cell viability of T24/Dox cells by CCK8 assay (Figure 2C, *P* < 0.05). Secondly, single label flow cytometry indicated T24/Dox cells with TUG1 knockdown showed a significant S phase block (Figure 2D, *P* < 0.05). Thirdly, TUG1 knockdown advanced cell apoptosis of T24/Dox cells by double label flow cytometry (Figure 2E, *P* < 0.05).

Thus, it was identified that knockdown of TUG1 inhibited the Dox resistance in T24/Dox cells.

### Knockdown of TUG1 restrained Wnt/β-catenin pathway

To discuss the regulatory mechanism of TUG1 on Dox resistance in BUC, the data of microarray assay was analyzed by bioinformatics. Gene Ontology (GO) analysis of lncRNAs differentially expressed between Dox (−) and Dox (+) BUC tissues indicated the major enriched processes were cell cycle and apoptosis. And Kyoto Encyclopedia of Genes and Genomes (KEGG) pathway analysis showed the most enriched pathway was Wnt/β-catenin pathway. Therefore, Wnt/β-catenin pathway was chosen as targets to highlight the TUG1 associated Dox resistance in BUC.

**Figure 3:**
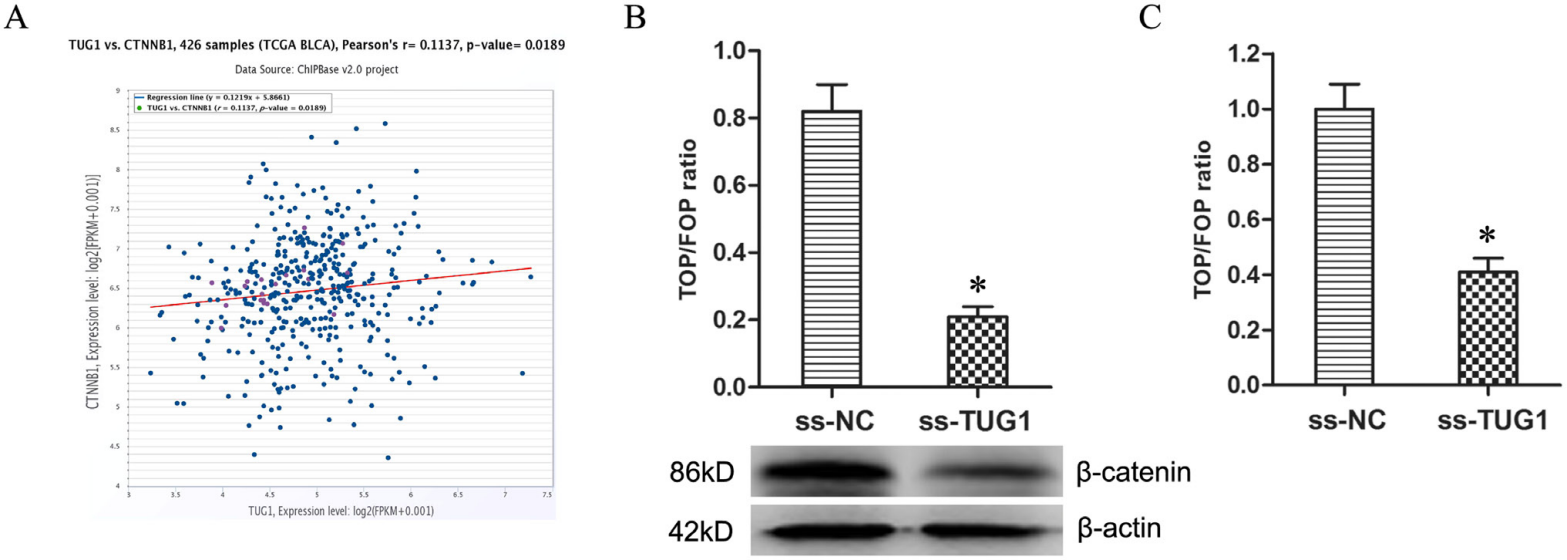
Knockdown of TUG1 restrained Wnt/β-catenin pathway (**A**) The co-expression patterns between TUG1 and β-catenin (CTNNB1) in BUC is searched using the online server ChIPBase. (**B**) The expression of β-catenin protein in T24/Dox cells. (**C**) The ratio of TOP/FOP values in T24/Dox cells examined by luciferase assay. *P < 0.05.

Firstly, the co-expression patterns (log2-scale) between TUG1 and β-catenin (CTNNB1) in TCGA Pan-Cancer (PANCAN) showed that the expression of TUG1 was positively related to the expression of β-catenin (Figure 3A), with a Pearson coefficient *r* = 0.1137 and *P* = 0.0189 (two tail, *t*-test) in 426 BUC samples. Secondly, TUG1 knockdown decreased the expression of β-catenin protein in T24/Dox cells (Figure 3B, *P* < 0.05). Thirdly, the TOP/FOP Flash luciferase reporter assay showed TUG1 knockdown depressed the TOP/FOP ratio in T24/Dox cells significantly (Figure 3C, *P* < 0.05), which represented the transcriptional activity of Wnt/β-catenin pathway.

Together, the data demonstrated TUG1 knockdown restrained the activity of Wnt/β-catenin pathway.

### Wnt/β-catenin pathway mediated TUG1-induced effecting on Dox resistance in 24/Dox cells

**Figure 4:**
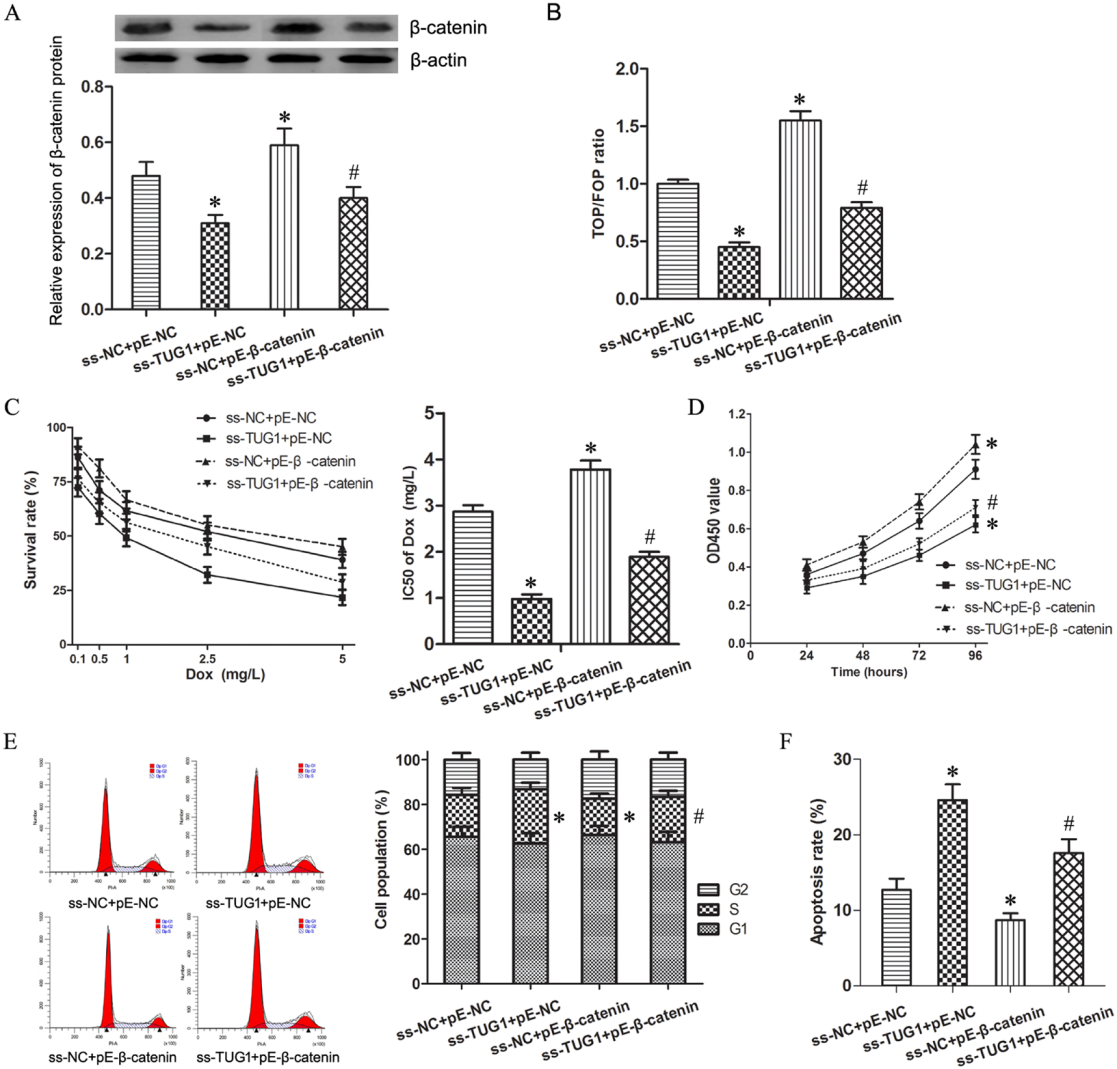
Wnt/β-catenin pathway mediated TUG1-induced effecting on Dox resistance in 24/Dox cells (**A**) The expression of β-catenin protein in T24/Dox cells. (**B**) The ratio of TOP/FOP values in T24/Dox cells examined by luciferase assay. (**C**) The dose-response curve and IC50 of T24/Dox cells to DOX. (**D**) The cell viability of T24/Dox cells detected by CCK8 assay. (**E**) The cell cycle of T24/Dox cells detected by flow cytometry. (**F**) The cell apoptosis of T24/Dox cells detected by flow cytometry. *P < 0.05 vs ss-NC+pE-NC, ^#^*P* < 0.05 vs ss-TUG1+pE-NC.

The expression vector for β-catenin (pE-β-catenin) was transfected into T24/DR cells to activate Wnt/β-catenin signaling which restrained by knockdown of TUG1. Western blotting and TOP/FOP Flash luciferase reporter assays indicated the Wnt/β-catenin pathway was activated significantly (Figure 4A and 4B, *P* < 0.05).

While combining use ss-TUG1 and pE-β-catenin, T24/Dox showed significantly increased Dox resistance when compared with ss-TUG1 group. The IC50 of Dox in T24/Dox cells was increased from 0.98 ± 0.16 mg/L to 1.89 ± 0.21 mg/L (Figure 4C, *P* < 0.05). In addition, under Dox treatment (0.5 mg/L), β-catenin enhancement promoted cell viability and cell division as well as suppressed cell apoptosis in T24/Dox cells (Figure 4D–4F, *P* < 0.05). However, ss-TUG1 and pE-β-catenin could not restore the Dox resistance to the original level of pE-β-catenin group.

Therefore, these results suggest that activation of Wnt/β-catenin signal pathway could partly restore the effecting on Dox resistance induced by TUG1 knockdown in T24/Dox cells.

## DISCUSSION

For clinicians, chemotherapy is an effective approach to prevent metastasis and recrudescence of BUC. Dox is the one of the most common and effective agent used widely in intravesical and systemic chemotherapy for BUC. Dox belongs to anthracycline antibiotic, which can integrate with nucleus DNA and damage DNA structure to make apoptosis and cell growth arrest [18]. However, Dox resistance had also been a barrier leading to failure treatment.

Recently, some lncRNAs had been reported to participate in the genesis of chemoresistance in almost all malignant tumors, including BUC. For instance, lncARSR advanced the Dox resistance in hepatocellular carcinoma (HCC), and might be a potential prognostic biomarker and therapeutic target for HCC [19]; lncRNA HOTAIR depressed the Dox sensitivity in BUC [20].

In our lncRNA array, TUG1 showed an over seven-fold up-regulation in Dox (−) BUC samples compared with Dox (+) BUC samples. And, the following qRT-PCR confirmed the up-regulation of TUG1 in BUC tissues and cell line resistant to Dox. The results revealed that the up-regulation of TUG1 was correlated with the poor response of BUC patients to Dox chemotherapy, which provided initial evidence that TUG1 played an important role in the Dox resistance of BUC.

Recent studies reported TUG1 was up-regulated in colorectal cancer resistant to methotrexate, knockdown of TUG1 re-sensitized the colorectal cancer cells to methotrexate [21]; high TUG1 expression was related to chemoresistance in esophageal squamous cell carcinoma to platinum combined with 5-fluorouracil or paclitaxel [22]. However, whether TUG1 is involved in BUC chemoresistance remains unknown, especially to Dox.

In order to validate the vital roles of TUG1 on Dox resistance, loss of function assays were applied to clarify the impacts of TUG1 on Dox resistance in BUC. Knockdown of TUG1 restrained the IC50 of T24/Dox cells to Dox, and advanced the Dox-induced cytotoxicity, which demonstrated that TUG1 knockdown inhibited Dox resistance in T24/Dox cells. However, the underlying mechanism remain unclear.

The data of microarray assay was analyzed by bioinformatics, and KEGG pathway analysis showed Wnt/β-catenin pathway was the most enriched pathway. And, TCGA Pan-Cancer (PANCAN) showed that the expression of TUG1 was positively related to the expression of β-catenin in BUC. Together with the published references [23], Wnt/β-catenin pathway was chosen as targets to highlight the TUG1 associated Dox resistance in BUC. It is generally known that Wnt/β-catenin pathway is closely involved in tumorigenesis, β-catenin is one of the important downstream effectors [24–27]. Fan Y found lncRNA UCA1 promoted cisplatin resistance of BC cells via Wnt/β-catenin pathway [28]. Then, western blotting and TOP/FOP Flash luciferase reporter assay were used to confirm that TUG1 knockdown restrained the activity of Wnt/β-catenin pathway.

Based on above findings, we put forward hypothesis that knockdown of lncRNA TUG1 might inhibit Dox resistance of BUC via Wnt/β-catenin pathway. Subsequent series of experiments confirmed that activating Wnt/β-catenin pathway by upregulating β-catenin reversed the effects of TUG1 knockdown on Dox resistance in T24/Dox cells, Wnt/β-catenin pathway partially mediated effecting induced by TUG1 knockdown in 24/DR cells.

In our previous study, knockdown of PVT1 gene could downregulate the expression of multidrug resistance protein 1 (MDR1) and multidrug resistance associated protein 1 (MRP1) expression in BUC cells through Wnt/β-catenin pathway, and then participate in the formation of chemoresistance to Dox and cisplatin [29]. Zhang ZM et al. found that Pygo2, a newly identified Wnt/β-catenin pathway component, activated the expression of MDR1 expression and mediated chemoresistance in breast cancer [30]. So, we considered MDR1 and MRP1 mediated the regulation of TUG1 on Dox chemoresistance in BUC.

In conclusion, high-expression of lncRNA TUG1 was related with the poor response of BUC patients to Dox chemotherapy, knockdown of TUG1 inhibited Dox resistance of BUC via Wnt/β-catenin pathway. Our findings elucidate a potential mechanism of BUC chemoresistance, and indicate TUG1 might be a novel potential therapeutic target in BUC.

## MATERIALS AND METHODS

### Cell culture and reagent treatment

Human BUC T24 cell lines were purchased from the American Type Culture Collection (Manassas, VA, USA). T24 cells were cultured Dulbecco’s Modified Eagle’s Medium (DMEM) supplemented with 10% fetal bovine serum (FBS; Gibco, Carlsbad, CA, USA), and maintained at 37°C in a humidified incubator with 5% CO_2_. The DOX resistant T24/Dox cell line was established in our laboratory previously via co-culture with continuously increasing DOX (Sigma-Aldrich, St. Louis, MO, USA) concentrations, which showed resistance to DOX [31].

### Clinical specimens

82 BUC specimens were gathered from the Department of Urinary surgery of Shengjing Hospital of China Medical University from Mar 2014 to Apr 2016 through cystoscope. All BUC patients were treated by chemotherapy with DOX, and the chemotherapy effects were evaluated in accordance with RECIST Response Evaluation Criteria. Patients resistant to DOX (−) were 51 cases, and patients sensitive to DOX (+) were 31 cases. This study was approved by the Ethics Committees of China Medical University, and the permissions of surgical patients were achieved.

### Quantitative real time PCR (qRT-PCR)

BUC tissues and cells were treated with TRIzol reagent (Invitrogen, Foster City, CA, USA) to extract total RNA. According to manufacturer’s instructions, the expression of TUG1 was detected by SYBR^®^ Green Master Mix Kit (Qiagen, Hilden, GER). The primers of TUG1 were 5′- CAAGAAACAGCAACACCAGAAG -3′ (forward) and 5′- TAAGGTCCCCATTCAAGTCAGT -3′ (reverse) [32]. The relative expression of TUG1 was normalized to the expression of β-actin and quantified with relative quantitative method.

### Cells transfection

The smart silencer for TUG1 (ss-TUG1) and negative control vector (ss-NC) were constructed by the Ribobio Corporation (Guangzhou, Guangdong, China). pEGFP-N1-β-catenin (pE-β-catenin) was constructed by cloning the full sequence of β-catenin into pEGFP-N1 vector, and empty pEGFP-N1was used as negative control (pE-NC).

According to manufacturer’s instructions, the T24/Dox cells were transfected with Lipofectamine 3000™ (Invitrogen, Foster City, CA, USA), and qRT-PCR was applied to examine the transfected efficiency. The stable cell line was established by treating with G418 (Sigma-Aldrich, St. Louis, MO, USA).

### Cell proliferation assay

The Cell Counting Kit-8 (CCK8) method was used to examine the cell proliferation ability. In details, T24/Dox cells were seeded in 96-well plate. Each well was added with 10 μl Cell Counting Kit-8 (Beyotime, Jiangsu, China), and incubated for 2 hours at 37°C. Absorbance at 450 nm was recorded using the SpectraMax M5 microplate reader (Molecular Devices, Sunnyvale, CA, USA) [33].

### Cell cycle detection

The cell cycle was analyzed by flow cytometry. In brief, 1 × 10^6^ cells were harvested and following the cells were fixed in 70% cold ethanol, pelleted and washed once with PBS and re-suspended in propidium iodide (PI) solution (50 μg/mL) for 30 minutes in the dark. Then, the cell cycles were determined by the FACScan Canto II flow cytometry (Becton Dickinson, USA) within 1 hour and the results were analyzed through the Diva 8.0 software.

### Apoptosis detection

The double label flow cytometry was used to detect the apoptosis rate with Annexin V-FITC apoptosis detection kit (Biosea, Beijing, China) according to manufacturer’s instructions. 1 × 10^6^ /ml cells were incubated with 2.5 μl FITC-Annexin V and 2.5 μl propridium iodide (PI) for 10 minutes in the dark at room temperature, and 400 μl binding buffer was added to terminate the reaction. The data was analyzed by FACSCanto II using the Diva 8.0 software (BD, USA). Cells at the right lower quadrant (FITC-Annexin V-positive and PI-negative) were defined as apoptosis.

### Chemotherapy resistance assay

Cells were treated with DOX at various concentrations (0.1 mg/L, 0.5 mg/L, 1 mg/L, 2.5 mg/L, 5 mg/L) 24 hours later. The cell viability was detected by CCK8 method 48 hours later. Based on the data, we charted the dose-response curve and calculated the half maximal inhibitory concentration (IC50) [29, 32].

### Western blotting

Protein (30 μg) extracted from T24/Dox cells were subjected to 10 % sodium dodecyl sulfate-polyacrylamide gels electrophoresis (SDS-PAGE) and transferred to a 0.22 μm polyvinylidene difluoride (PVDF) membrane (Millipore, MA). The PVDF membrane was blocked and incubated with β-catenin antibodies (ab32572, Abcam, USA) at 4°C overnight. Then, the membrane was incubated with second antibody and treated with BeyoECL Plus reagent (Beyotime, Nanjing, China). The image was scanned and analyzed with Image J (National Institutes of Health, USA) by normalized to the inference gene of β-actin.

### Luciferase assay

The TOP Flash and FOP FLASH luciferase reporter vectors which contain wild-type or mutant TCF binding sites were purchased from Biovector NTCC Ltd (Beijing, China). T24/Dox cells were transiently co-transfected with luciferase plasmid and ss-TUG1 vector using Lipofectamine™ 3000. 48 hours later, dual-lucy assay kit from Vigorous Biotech (Beijing, China) was used to quantified the activity of Wnt/β-catenin pathway according to manufacturer’s instructions [34]. The firefly luciferase used as a base line and renilla luciferase used as the internal control.

### Statistical analysis

All data were showed as means ± SD of five independent experiments and analyzed with GraphPad Prism 5.0 (Graphpad Software, La Jolla, CA). The data were analyzed using Student’s *t*-test and one-way ANOVA. A *P*-value of less than 0.05 was considered to be statistically significant and indicated by (^*^ and ^#^).
